# Intrahepatic cholangiocarcinoma with a tumor thrombus extending from the inferior vena cava to the right atrium: a case report

**DOI:** 10.1186/s40792-020-01085-4

**Published:** 2021-01-06

**Authors:** Genya Hamano, Shigekazu Takemura, Shogo Tanaka, Hiroji Shinkawa, Takanori Aota, Hiromichi Fujii, Takashi Murakami, Yuko Kuwae, Shoji Kubo

**Affiliations:** 1 Department of Hepato-Biliary-Pancreatic Surgery, Osaka City University Graduate School of Medicine, 1-4-3 Asahimachi, Abeno-ku, Osaka, 545-8585 Japan; 2 Department of Cardiovascular Surgery, Osaka City University Graduate School of Medicine, 1-4-3 Asahimachi, Abeno-ku, Osaka, 545-8585 Japan; 3 Department of Pathology, Osaka City University Graduate School of Medicine, 1-4-3 Asahimachi, Abeno-ku, Osaka, 545-8585 Japan

**Keywords:** Intrahepatic cholangiocarcinoma, Tumor thrombus, Inferior vena cava, Liver resection, Thrombectomy

## Abstract

**Background:**

Vascular invasion involving a tumor thrombus in the inferior vena cava and/or right atrium is an unfavorable prognostic factor after intrahepatic cholangiocarcinoma resection. We report an intrahepatic cholangiocarcinoma case with a tumor thrombus extending from the left hepatic vein via the inferior vena cava to the right atrium.

**Case presentation:**

A 58-year-old man with epigastralgia was referred to our hospital after an emergent transcatheter arterial embolization was done following the radiological diagnosis of a ruptured hepatic tumor. The serum concentrations of carcinoembryonic antigen, carbohydrate 19-9, duke pancreatic monoclonal antigen type 2, and cytokeratin-19 fragments were elevated; meanwhile those of alfa-fetoprotein and des-γ-carboxy prothrombin were within normal ranges. A contrast-enhanced computed tomography scan showed a heterogeneously enhanced tumor, 13 cm in diameter, in the left lobe of the liver, enlarged lymph nodes along the lesser curvature of the stomach, and a tumor thrombus extending from the left hepatic vein via the inferior vena cava to the right atrium. We performed a left hemihepatectomy and tumor thrombectomy under total hepatic vascular exclusion to reduce the risk of sudden death. After dissection of the liver parenchyma along the left side of the middle hepatic vein, except for the left hepatic vein, the inferior vena cava just below the right atrium could be clamped by pulling down the left lobe of the liver toward the caudal side. The thrombus could be removed by incising the inferior vena cava under total hepatic vascular exclusion. Microscopic examination showed a tubular adenocarcinoma. Immunohistochemical staining was positive for cytokeratin-7, cytokeratin-19, and epithelial membrane antigen, but negative for arginase-1, glypican-3, and hepatocyte. The patient was pathologically diagnosed with an intrahepatic cholangiocarcinoma with a tumor thrombus in the inferior vena cava. Adjuvant chemotherapy with tegafur/gimeracil/oteracil was administered for 1 year. The patient remained in good health without cancer recurrence for over 4 years after the operation.

**Conclusion:**

An aggressive surgical approach may be indicated for intrahepatic cholangiocarcinoma with a tumor thrombus in the inferior vena cava and/or right atrium to avoid the risk of impending death.

## Background

Previous studies have shown that intrahepatic metastasis, lymph node metastasis, periductal invasion, vascular invasion, and positive surgical margin are risk factors associated with poor prognosis after surgical treatment for intrahepatic cholangiocarcinoma (ICC) [1–5]. Although ICC is sometimes associated with hepatic vein or inferior vena cava (IVC) invasion or a thrombus in the IVC and/or the right atrium (RA), only few patients have undergone surgical resection for IVC and/or RA thrombus [3, 4]. Herein, we report a resected case of ICC with a tumor thrombus extending from the IVC to the RA.

## Case presentation

A 58-year-old man complaining of epigastralgia was admitted to a hospital. The patient had a left empyema which was drained when he was 48 years. At the time of admission, he was receiving pharmacological treatment for diabetes mellitus and hypertension. After an emergent transcatheter arterial chemoembolization following the diagnosis of a ruptured hepatic tumor, he was referred to our hospital for surgical treatment.

On admission, a hard elastic mass was palpable in his upper abdomen. Liver function test results were within normal ranges (Table 1). Tests for serum anti-hepatitis C virus antibody, hepatitis B surface antigen, and hepatitis B core antibody were negative. Although serum concentrations of alpha-fetoprotein and des-γ-carboxy prothrombin were within normal ranges, those of carbohydrate antigen 19-9, carcinoembryonic antigen, duke pancreatic monoclonal antigen type 2, s-pancreas antigen-1, and cytokeratin-19 fragments were elevated.

**Table 1:** Laboratory test results on admission

WBC	6100/μl	Total protein	7.5 g/dl	HCV-Ab	(−)
RBC	442 × 10^4^/μl	Albumin	3.7 g/dl	HBs-Ag	(−)
Hemoglobin	12.5 g/dl	C-reactive protein	0.41 mg/dl	HBc-Ab	(−)
Hematocrit	37.4 %	BUN	14 mg/dl	CEA	8.2 ng/ml
Platelet count	23.5 × 10^4^/μl	Creatinine	0.72 mg/dl	CA19-9	3600 U/ml
Prothrombin activity	83 %	Sodium	138 mEq/l	Alpha-fetoprotein	16.2 ng/ml
Total bilirubin	0.7 mg/dl	Potassium	4.3 mEq/l	DCP	12 mAU/ml
AST	17 U/l	Chloride	103 mEq/l	DUPAN-2	12000 U/ml
ALT	13 U/l	Hemoglobin A1c	7.6 %	SPan-1	190 U/ml
γ-GTP	62 U/l	ICGR15	27.2 %	CYFRA	22.4 ng/ml

*WBC* white blood cell count, *RBC* red blood cell count, *AST* aspartate aminotransferase, *ALT* alanine aminotransferase, *γ-GTP* *γ*-glutamyl transpeptidase, *BUN* blood urea nitrogen, *ICGR15* indocyanine green retention rate at 15 min, *HCV-Ab* hepatitis C virus antibody, *HBs-Ag* hepatitis B surface antigen, *HBc-Ab* hepatitis B core antibody, *CEA* carcinoembryonic antigen, *CA19-9* carbohydrate antigen 19-9, *DCP* des-*γ*-carboxy prothrombin, *DUPAN-2* duke pancreatic monoclonal antigen type 2, *Span-1* s-pancreas antigen-1, *CYFRA* cytokeratin 19 fragments

**Fig. 1:**
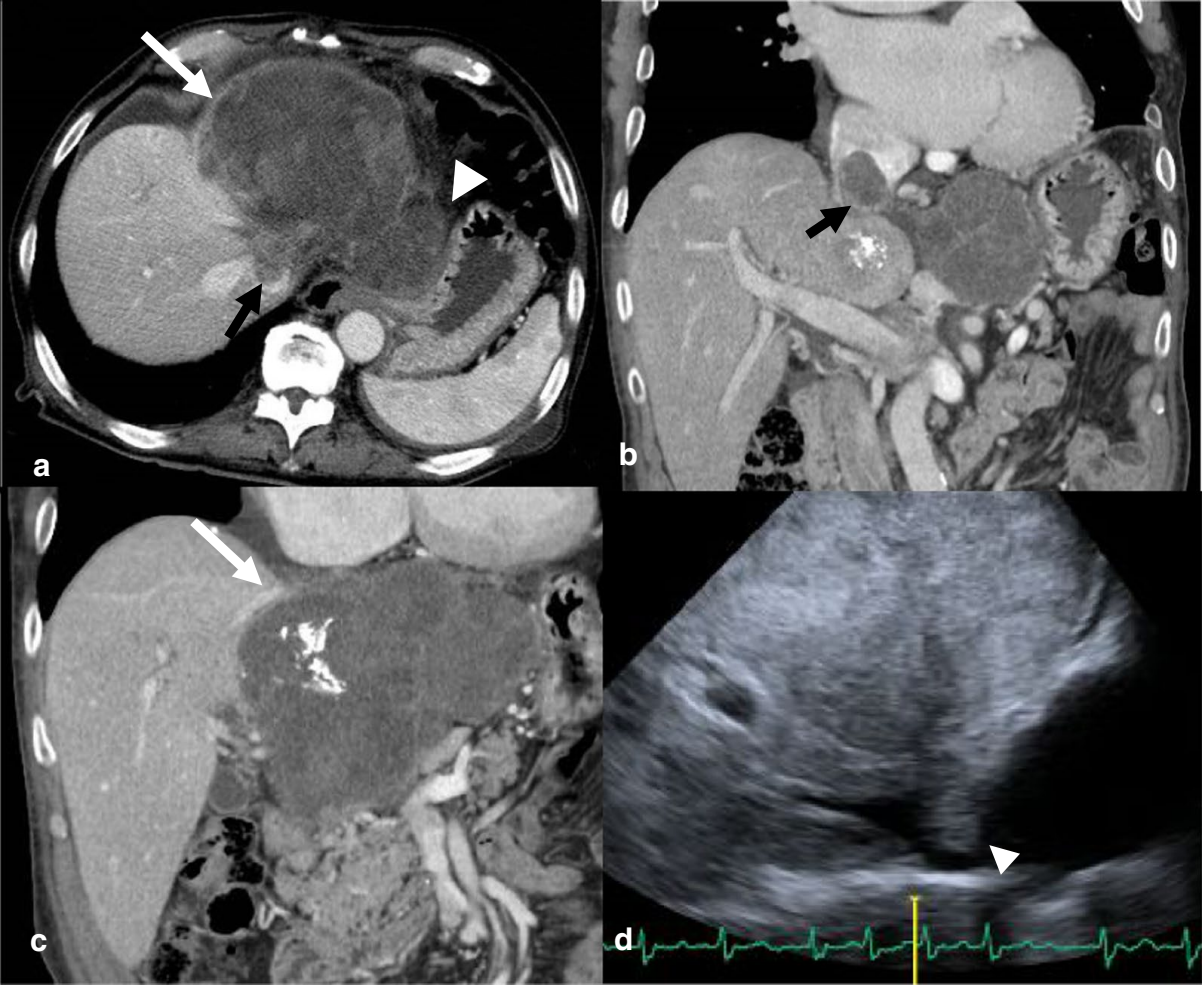
Diagnostic imaging. **a** Enhanced computed tomography scan showing a 13-cm tumor in the left lobe of the liver (long white arrow), with a tumor thrombus arising from the left hepatic vein into the inferior vena cava (short black arrow), and enlarged lymph nodes (white arrow head). **b** Coronal section image showing the tumor thrombus extending from the left hepatic vein via the inferior vena cava to the right atrium (short black arrow). **c** The main tumor was seemingly attached to the middle hepatic vein, but did not involve it (white arrow). **d** Transthoracic echocardiography showing a tumor thrombus extending from the inferior vena cava to the right atrium (short white arrow)

Contrast-enhanced computed tomography scan showed a heterogeneously enhanced tumor, 13 cm in diameter, occupying the left lobe of the liver, and an intraluminal mass extending from the left hepatic vein (LHV) via the IVC to the RA. The main tumor was seemingly attached to the middle hepatic vein. Some enlarged lymph nodes (6 cm in the largest diameter) were detected along the lesser curvature of the stomach (Fig. 1a–c). Transthoracic echocardiography showed the cranial side of the tumor thrombus just inside the RA (Fig. 1d). We diagnosed a mass-forming type ICC or mixed hepatocellular and cholangiocarcinoma with lymph node metastasis and a tumor thrombus extending from the LHV via the IVC to the RA.

Although curative resection was difficult due to lymph node metastasis and tumor thrombus in the IVC, we thought that surgical treatment was necessary, given the risk of impending and sudden death by cardiopulmonary failure with rapid progression. As a result, we performed a left hemihepatectomy, cholecystectomy, lymph node dissection, and tumor thrombectomy, while considering an open cardiac surgery under cardiopulmonary bypass. We also prepared to reconstruct the IVC with an autologous or bovine pericardial patch and a ring-reinforced expanded polytetrafluoroethylene graft. The predicted remnant liver volume was 91% of the total liver volume, excluding the tumor volume; it was evaluated using 3D simulated liver volumetry.

**Fig. 2:**
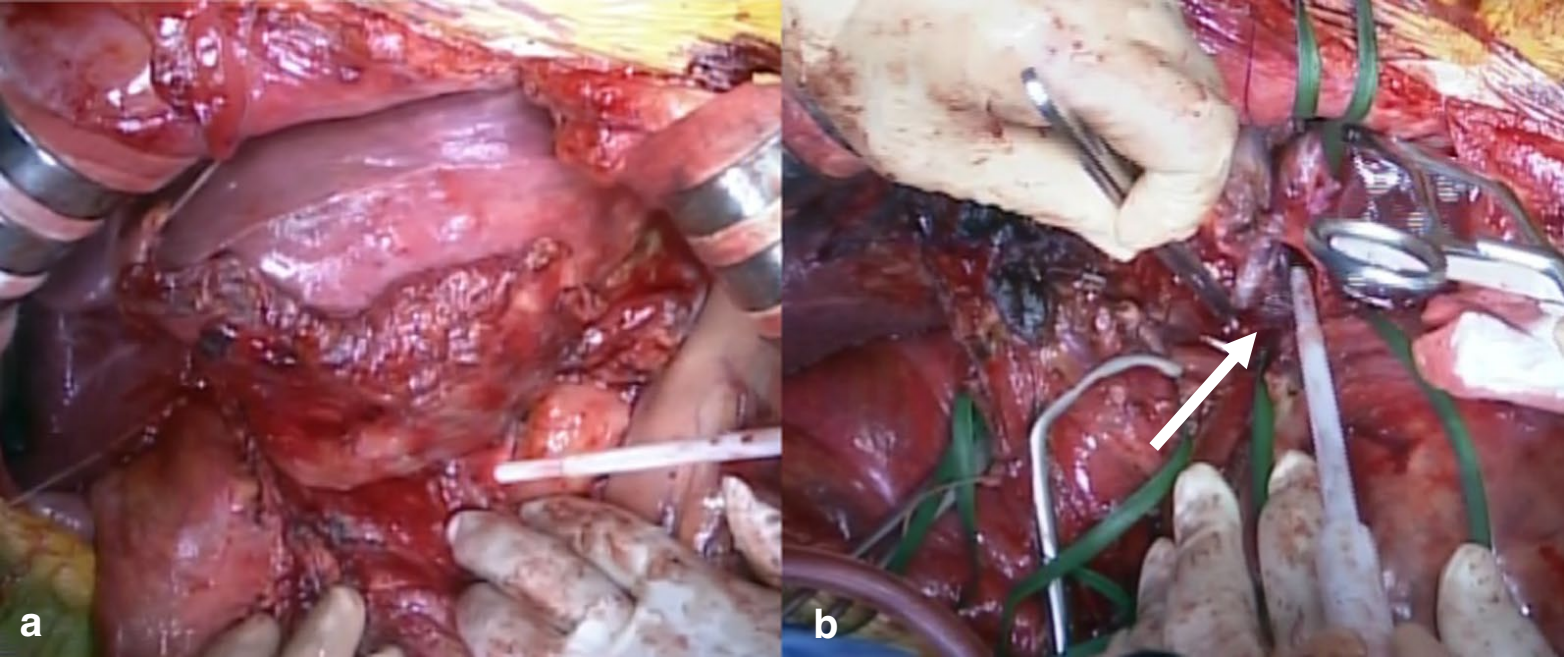
Intraoperative view. **a** A tumor with the size of an infant head is shown in the left lobe of the liver. The tumor adhered to enlarged lymph nodes along the lesser curvature of the stomach. **b** After the liver transection, the tumor thrombus (white arrow) was removed from the inferior vena cava by total hepatic vascular exclusion of the liver

During the operation, although an intermediate amount of serous ascites was present, there were no macroscopic signs of peritoneal dissemination. A tumor with the size of an infant’s head in the left lobe of the liver was confirmed; it was surrounded by the greater omentum and adhered to enlarged lymph nodes along the lesser curvature of the stomach, hepatoduodenal ligament, mesentery of the transverse colon, and diaphragm (Fig. 2a). Intraoperative transesophageal echocardiography demonstrated that a tumor thrombus was present in the IVC and the cranial side of the tumor thrombus reached the RA entry. Although the CT scan showed that the main tumor was seemingly attached to the middle hepatic vein, the tumor did not involve this vein, and transesophageal echocardiography revealed that the middle hepatic vein was completely separated from the tumor.

The accessory left hepatic artery and left hepatic artery were ligated and divided, followed by the left portal vein which was ligated. Lymph nodes in the hepatoduodenal ligament were dissected. After expanding the hepatic hilar region by dissecting the liver parenchyma along the left side of the middle hepatic vein, we cut the left portal vein and left hepatic duct. We then did an en bloc resection of the liver parenchyma with enlarged lymph nodes along the lesser curvature of the stomach. As a result, only the LHV-involving tumor thrombus was connected to the IVC. We ligated and cut the bilateral subphrenic veins and dissected the diaphragm vertically, opening the pericardial membrane to expose the IVC to the RA. The IVC was encircled proximally to the RA in the pericardial cavity. We then evaluated the extent of the tumor thrombus by transesophageal echocardiography again. It was possible to clamp the IVC at the cranial site of the tumor thrombus (proximal to the RA), as the tumor thrombus could be moved by pulling the left lobe of the liver toward the caudal side. The blood pressure during clamping of the IVC could be maintained by administration of transfusion and catecholamines. During total hepatic vascular exclusion (THVE), we opened the common trunk of the LHV and IVC. We performed a tumor thrombectomy by resecting the intraluminal membrane of the IVC at the site where the tumor thrombus intensively adhered to the IVC and washed inside the IVC (Fig. 2b). The postoperative transesophageal echocardiography showed no residual tumor thrombus. After side-clamping the IVC, THVE was stopped for 10 min. The LHV was cut, and the left lobe of the liver was removed. Finally, the stump of the left hepatic vein was sutured. The pericardium and diaphragm were sutured roughly. The operative time was 645 min. Intraoperative blood loss was 6800 ml.

**Fig. 3:**
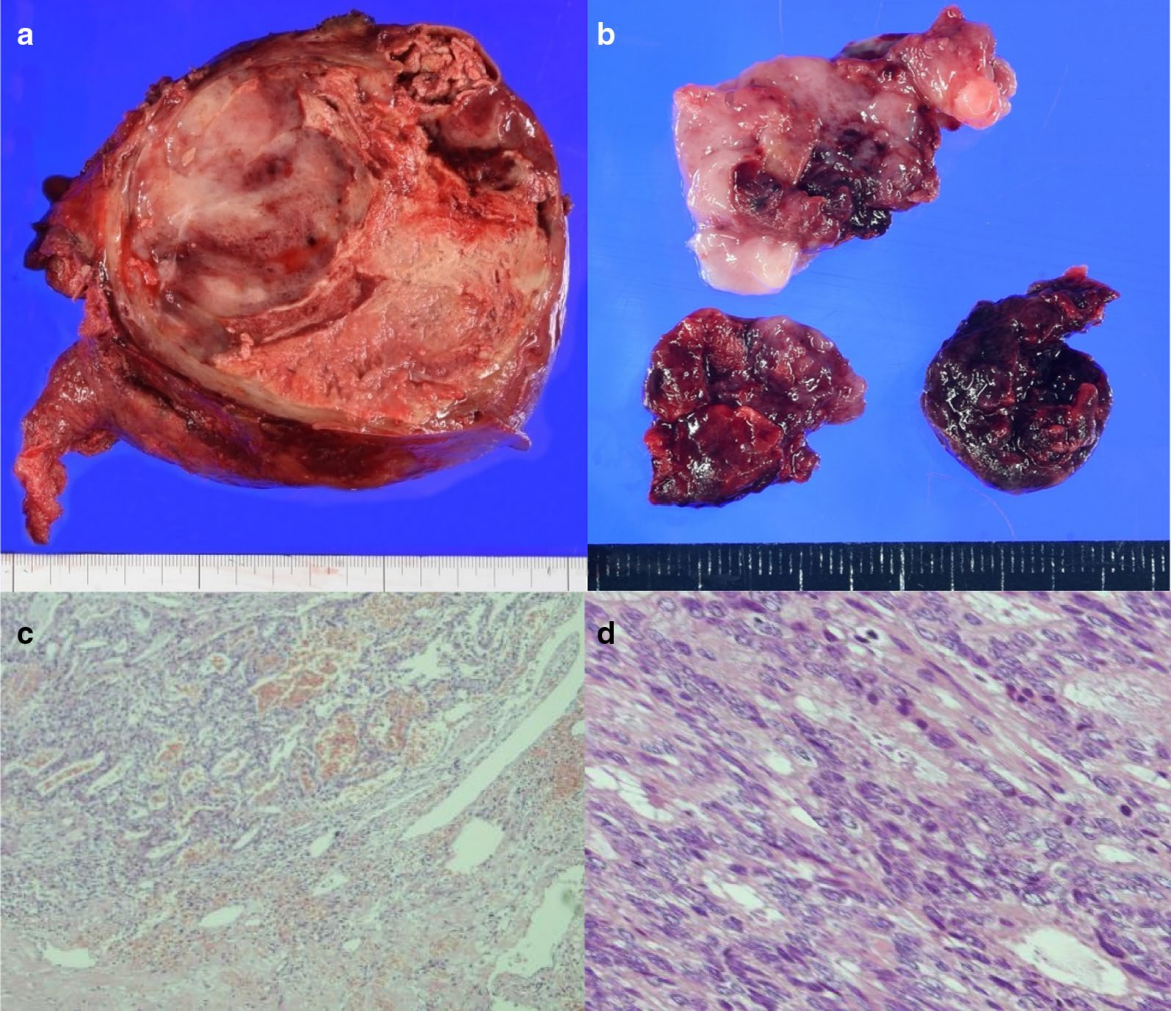
Operative specimens and pathological findings. **a** The cut surface of the resected specimen shows a tumor with hemorrhagic necrotic changes, 13 × 12 cm in size. **b** Tumor thrombus in the inferior vena cava. **c** Tumor cells were moderately differentiated adenocarcinoma (hematoxylin and eosin staining, × 40) and **d** adenocarcinoma (HE, × 200)

The resected liver weighed 1450 g. The resected liver tumor was white, with a 13 × 12-cm diameter and fibrous capsule, and with small intrahepatic metastases (Fig. 3a, b). The enlarged lymph nodes at the lesser curvature of the stomach adhered to the tumor.

**Fig. 4:**
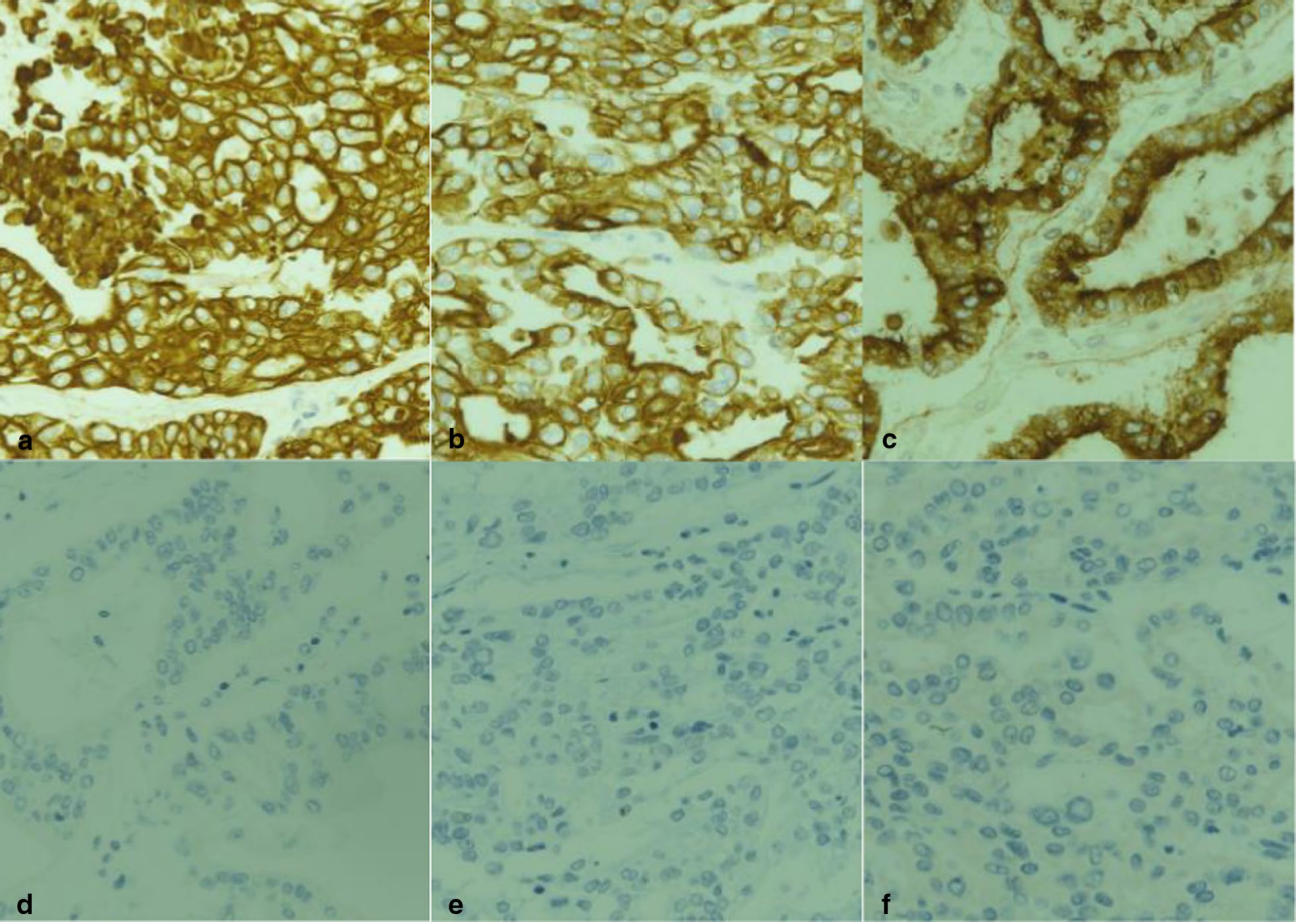
Immunohistochemical findings. Immunohistochemical analysis revealed that tumor cells were positive for cytokeratin-7 (**a**), cytokeratin-19 (**b**), and epithelial membrane antigen (**c**), but negative for arginase-1 (**d**), glypican-3 (**e**), and hepatocyte (**f**)

Microscopic examination of the resected tumor showed that the main tumor was a moderately differentiated tubular adenocarcinoma (Fig. 3c, d) and that cancer cells infiltrated the capsule and portal vein around the tumor. Intrahepatic metastatic nodules were detected near the main tumor. The pathological examination of the noncancerous lesion revealed that the fatty change within the hepatocyte was not apparent, and the fibrous change of the perisinusoidal area was mild. Immunohistochemical staining revealed that the cancer cells were positive for cytokeratin-7, cytokeratin-19, and epithelial membrane antigen, but negative for arginase-1, glypican-3, and hepatocyte: these are immunohistochemical markers of hepatocytes (Fig. 4). Based on these findings, we confirmed the diagnosis of a mass-forming type ICC with lymph node metastasis and a tumor thrombus extending from the LHV via the IVC to the RA. The ICC was classified as T3N1M0 Stage IIIB, according to the UICC classification system [6]. According to the Liver Cancer Study Group of Japan classification [7], the ICC was classified as T4N1M0 (vp1 and vv3) Stage IVB.

Although conservative treatment with medication was needed for postoperative delayed gastric emptying, the patient was discharged on the 41st postoperative day. Adjuvant chemotherapy with tegafur/gimeracil/oteracil was started 1 month after the operation and continued for the following 1 year. The patient remained in good health without cancer recurrence 4 years after the operation.

## Discussion

This patient underwent liver resection and thrombectomy for mass-forming type ICC with a tumor thrombus extending from the IVC to RA. To the best of our knowledge, based on a thorough literature search in PubMed with the key words such as intrahepatic cholangiocarcinoma, tumor thrombus, hepatectomy (liver resection), thrombectomy, inferior vena cava, and right atrium, this is the first report of a successfully resected case of ICC with a tumor thrombus extending from the LHV via the IVC to the RA.

Patients with a tumor thrombus in the IVC or RA and a hepatic tumor such as hepatocellular carcinoma and ICC have a high mortality risk, as a tumor thrombus can lead to pulmonary embolism or occlusion of the tricuspid valve, lung metastasis, pulmonary infarction, secondary Budd–Chiari syndrome, ball-valve thrombus syndrome, and heart failure [8–10]. The risk for cardiopulmonary collapse is high and heart failure or sudden death is noted as the cause of death in 25% of the patients with HCC with intracavitary cardiac involvement [11]. From our preoperative evaluation, we thought that this was an oncologic emergency. Although transarterial chemoembolization or radiation therapy is thought to be better for local control and effective for tumor down-staging in HCC cases with a tumor thrombus in the IVC, no effective treatment has been reported as the preoperative therapy for ICC with a tumor thrombus in the IVC. In this patient, with a good liver function and no comorbidities, we decided to perform a liver resection with thrombectomy for ICC to prevent sudden death.

According to a nationwide follow-up survey of primary liver cancer patients [4], of 808 patients with ICC, 60 patients (7.4%) had a major hepatic vein invasion and 20 patients (2.5%) had IVC or RA invasion. Of 580 patients who underwent surgical treatment, 42 patients (7.2%) had a major hepatic vein invasion, and 9 patients (1.6%) had IVC or RA invasion. Cases of ICC with IVC invasion are classified as either direct invasion to the IVC or a tumor thrombus in the IVC. However, this nationwide survey did not distinguish between these types of invasion [4]. In our department, of 123 patients who underwent liver resection for ICC between 1998 and 2019, 11 patients had invasion of the peripheral hepatic vein, 2 patients had invasion of the major hepatic vein, and only 1 patient had direct IVC invasion, while another patient (described in this case report) had a tumor thrombus extending from the LHV via the IVC to the RA. Studies with a larger sample size are required to discuss the differences between the clinicopathological characteristics of tumor thrombi in patients with HCC and those with ICC.

Although there have been recent advances in the surgical technique and equipment for liver resection with IVC or RA thrombectomy for liver tumors [12, 13], this procedure remains challenging and risky. Li et al. [9] reported a classification system for tumor thrombi in the IVC or RA, which includes three categories based on the anatomic location. A tumor thrombus in the IVC below the diaphragm is classified as type I (inferior hepatic type). A tumor thrombus in the IVC above the diaphragm, but still outside the RA is classified as type II (superior hepatic type), and a tumor thrombus above the diaphragm that enters the RA is classified as type III (intracardiac type). For type II, the intrathoracic IVC is approached via the abdominal cavity with an incision in the diaphragm with THVE. For type III, hepatectomy with thrombectomy is generally performed under cardiopulmonary bypass. Therefore, the surgical risk associated with type III is significantly higher than that associated with type II.

In the present case, the tumor thrombus was classified as type III based on preoperative diagnostic imaging, after which we have made preparations for the cardiopulmonary bypass. We determined that the extent of the IVC wall required to be resected would be limited when resecting the tumor thrombus. If reconstruction of the IVC was needed, we planned on doing so with an autologous or bovine pericardial patch, and we prepared a ring-reinforced expanded polytetrafluoroethylene graft if a longer length of the IVC needed to be reconstructed. However, as the tumor thrombus was present just inside the RA, we thought that it was possible to clamp the IVC just below the RA if the tumor thrombus did not adhere firmly to IVC and was pulled down with the left liver lobe toward the caudal side. In fact, during the operation, after resection of the left lobe of the liver except the hepatic vein, we were able to pull the thrombus as described, clamp the IVC, and remove the tumor thrombus under THVE without cardiopulmonary bypass. This approach may be useful when a tumor thrombus extends only inside the RA.

The prognosis after liver resection and thrombectomy for ICC with a tumor thrombus in the IVC and RA remains unclear due to the few patients who undergo this procedure. However, in general, the prognosis for such patients is poor due to the high risk of postoperative recurrence [8]. In this patient, the tegafur/gimeracil/oteracil regimen was administered for 1 year post-operatively as adjuvant chemotherapy, and cancer recurrence was not detected for 4 years after the operation. Future studies should investigate the usefulness of multidisciplinary treatments, including chemotherapy, which may be necessary for patients with advanced ICC.

## Conclusions

We performed liver resection with tumor thrombectomy for ICC with a tumor thrombus extending from the LHV via the IVC to the RA, preventing patient death and achieving a good prognosis. An aggressive surgical approach may be indicated for such ICCs with a tumor thrombus in the IVC and/or RA.

## Data Availability

Datasets supporting the presented conclusions are included within the article.

## Abbreviations

ICC: Intrahepatic cholangiocarcinoma
IVC: Inferior vena cava
RA: Right atrium
LHV: Left hepatic vein
THVE: Total hepatic vascular exclusion
